# Supplementary material for: Tenectin recruits integrin to stabilize bouton architecture and regulate vesicle release at the Drosophila neuromuscular junction
Source: eLife. 2018 Jun 14;7:e35518. doi: 10.7554/eLife.35518 (PMC6040883; doi:10.7554/eLife.35518)
Supplement: Transparent reporting form [file elife-35518-transrepform.docx]

***eLife’s* transparent reporting form**

We encourage authors to provide detailed information *within their submission* to facilitate the interpretation and replication of experiments. If you have any questions, please contact us: [editorial@elifesciences.org](mailto:editorial@elifesciences.org).

**Sample-size estimation**

- You should state whether an appropriate sample size was computed when the study was being designed
- You should state the statistical method of sample size computation and any required assumptions
- If no explicit power analysis was used, you should describe how you decided what sample (replicate) size (number) to use

Please outline where this information can be found within the submission (e.g., page numbers or figure legends), or explain why this information doesn’t apply to your submission:

A large sample size, as appropriate for each analysis, was computed and recorded in the following sections:

- electrophysiological recordings (page 8/Figure 2, pages 9-10/ Figure 3)

- quantification of various signal intensities in stacks of confocal images (Figures 1, 4, 5, 6, 7 and 8 and their correspondent supplemental data)

- quantification of electron micrographs (Figure 5)

- page 5, genetic screening and animal lethality

- bouton number and area (Figures 2- S1, 5, 7, and 8).

The total number of NMJs, animals or boutons examined is indicated under each bar as specified in each figure legend.

**Replicates**

- You should report how often each experiment was performed
- You should include a definition of biological versus technical replication
- The data obtained should be provided and sufficient information should be provided to indicate the number of independent biological and/or technical replicates
- If you encountered any outliers, you should describe how these were handled
- Criteria for exclusion/inclusion of data should be clearly stated
- High-throughput sequence data should be uploaded before submission, with a private link for reviewers provided (these are available from both GEO and ArrayExpress)

Please outline where this information can be found within the submission (e.g., page numbers or figure legends), or explain why this information doesn’t apply to your submission:

Each immunohistochemistry experiment was performed at least three times (as indicated at page 27). Every time, a set of NMJ confocal stacks (primarily muscle 4, abdominal segment A3) were selected and quantified. The total number of NMJs examined is indicated under each bar, as specified in all figure legends.

The Western blots and cell culture experiments were repeated four times or more.

**Statistical reporting**

- Statistical analysis methods should be described and justified
- Raw data should be presented in figures whenever informative to do so (typically when N per group is less than 10)
- For each experiment, you should identify the statistical tests used, exact values of N, definitions of center, methods of multiple test correction, and dispersion and precision measures (e.g., mean, median, SD, SEM, confidence intervals; and, for the major substantive results, a measure of effect size (e.g., Pearson's r, Cohen's d)
- Report exact p-values wherever possible alongside the summary statistics and 95% confidence intervals. These should be reported for all key questions and not only when the p-value is less than 0.05.

The statistical analyses for electrophysiological recordings were performed with ANOVA. Student’s t-test was used for comparing data generated by confocal and electron microscopy. The detailed information about analysis has been described in the Materials and Methods (pages 28- 29) and has been included in the main text for figure description as well as in figure legends.

Please outline where this information can be found within the submission (e.g., page numbers or figure legends), or explain why this information doesn’t apply to your submission:

(For large datasets, or papers with a very large number of statistical tests, you may upload a single table file with tests, Ns, etc., with reference to page numbers in the manuscript.)

**Additional data files (“source data”)**

- We encourage you to upload relevant additional data files, such as numerical data that are represented as a graph in a figure, or as a summary table
- Where provided, these should be in the most useful format, and they can be uploaded as “Source data” files linked to a main figure or table
- Include model definition files including the full list of parameters used
- Include code used for data analysis (e.g., R, MatLab)
- Avoid stating that data files are “available upon request”

Please indicate the figures or tables for which source data files have been provided:
